# Change in public awareness of symptoms and perceived barriers to seeing a doctor following Be Clear on Cancer campaigns in England

**DOI:** 10.1038/bjc.2015.32

**Published:** 2015-03-03

**Authors:** E Power, J Wardle

**Affiliations:** 1 Cancer Research UK, Angel Building, 407 St John Street, London EC1V 4AD, UK; 2 Department of Epidemiology and Public Health, Health Behaviour Research Centre, University College London, 1-19 Torrington Place, London WC1E 6BT, UK

**Keywords:** cancer awareness, barriers, primary care, symptoms, help-seeking

## Abstract

**Background:**

English Be Clear on Cancer (BCOC) campaigns aim to promote early presentation of potential cancer symptoms by (i) giving information on symptoms to look out for, and (ii) emphasising the approachability of the general practitioner (GP). This study examined public awareness of the targeted symptoms and perceived approachability of the GP before and after the national bowel and lung campaigns.

**Methods:**

The Cancer Research UK Cancer Awareness Measure (CAM) was included in the Opinions and Lifestyle survey (known then as the ‘Opinions Survey') run by the Office for National Statistics in October and November 2010 and 2012. Change in awareness of symptoms and barriers to help-seeking related to those targeted in the campaigns between the 2010 and 2012 surveys, was compared with change in awareness of symptoms and barriers not targeted by the campaigns.

**Results:**

Recall of ‘persistent cough' or ‘hoarseness' as a sign of cancer increased from 18% in 2010 to 26% in 2012 (*P*<0.001), and ‘change in bowel/bladder habits' increased from 21% to 43% (*P*<0.01). Recognition of these symptoms (from a list of symptoms) also increased significantly (both *P-*values <0.01). Awareness of non-targeted symptoms did not increase (all *P*-values >0.02). Barriers to visiting the GP targeted in the campaign (the doctor would be difficult to talk to and being worried about wasting the doctor's time) did not change, although several non-targeted barriers reduced.

**Conclusions:**

BCOC campaigns run in England in 2012 were associated with increased public awareness of some key symptoms of lung and bowel cancer. Barriers to visiting the GP that were targeted in the campaign were not reduced, indicating that a different approach may be needed to shift public attitudes towards GPs.

A major programme of work aiming to promote earlier diagnosis of cancer and ensure access to optimal treatment has been coordinated and supported by the National Awareness and Early Diagnosis Initiative (NAEDI): a public and third sector partnership in England, UK. One of the key areas targeted is public awareness of potential cancer signs and symptoms. Recall of potential symptoms has been shown to be low (Robb *et al*, 2009; Power *et al*, 2011; Simon *et al*, 2012; Low *et al*, 2013; Brain *et al*, 2014), and lower awareness has been linked with a longer intended delay in visiting a doctor with potential cancer symptoms (Simon *et al*, 2010; Quaife *et al*, 2014). Another key area is barriers to visiting the general practitioner (GP). Several studies have suggested that people can be reluctant to visit the GP with what may seem like minor symptoms (Macleod *et al*, 2009). Worry about ‘wasting the doctor's time' has been highlighted as a particularly strong barrier in the United Kingdom (Forbes *et al*, 2013).

As part of NAEDI, there have been a series of campaigns under the Be Clear on Cancer (BCOC) banner (http://campaigns.dh.gov.uk/category/beclearoncancer/), designed to improve public awareness of specific cancer signs and symptoms, and encourage prompt help-seeking, using a combination of TV, radio, press and other channels, depending on the level of roll out (national, regional or local). General practitioners feature prominently, with images of a friendly looking doctor alongside messages emphasising their approachability, such as ‘just tell me' or ‘your doctor wants to know'. The first campaign that was run nationally focused on bowel cancer and took place between January and March 2012. It aimed to raise awareness of change in bowel habits and blood in stool (using colloquial terminology) as possible cancer symptoms, and encouraged people with these symptoms to see their GP. This was followed by a national campaign on lung cancer in May and June 2012, which focused on the symptom of a persistent cough. The bowel campaign was then repeated in August and September 2012, although with less media spend than the first campaign. Both campaigns targeted those from lower socioeconomic groups aged over 50 (55+ for the bowel campaign) and their key influencers (friends and family). This paper focuses on these campaigns that took place in 2012, although there have been other campaigns run at a national level since then.

We used existing data from the validated Cancer Research UK Cancer Awareness Measure (CAM) (Stubbings *et al*, 2009) administered in national population-representative surveys carried out in late 2010 and 2012 to test the prediction that there would be greater awareness of the symptoms highlighted in these national campaigns than non-targeted symptoms, following the advertising. We also tested the hypothesis that barriers associated with the ‘approachability' of the GP would be reduced. Secondary analyses assessed differential change by gender, age, socioeconomic deprivation and ethnicity.

## Materials and methods

### Participants

Items from the CAM (Robb *et al*, 2009) were added to the Office for National Statistics (ONS) Opinions and Lifestyle survey (known then as the ‘Opinions Survey') for 2 months in the Autumn of 2010 and 2012. The ONS survey recruits using random probability sampling stratified by region, the proportion of households with no car, the proportion of households classified as NS-SEC categories 1–3 (managerial, professional and intermediate occupations) and the proportion of people aged over 65 years. Households are randomly selected from the Royal Mail's Postcode Address File of ‘small users', and individuals from each household are selected using a Kish grid. Potential respondents receive a letter providing a brief explanation of the purpose of the survey and advising them that an interviewer will be calling. A book of six first-class postage stamps is included with the letter as an incentive to maximise response. The survey is administered using face-to-face, computer-assisted interviews and is carried out in respondents' homes. All responses were anonymised; therefore, ethical approval was not required.

### Measures

Awareness of warning signs and symptoms was assessed first using an unprompted question to test symptom recall (‘There are many warning signs and symptoms of cancer. Please name as many as you can think of'). Responses matching those on the symptom list used for the prompted question (see below) were counted as correct. This was followed by a prompted question to test symptom recognition (‘The following may or may not be warning signs for cancer. We are interested in your opinion'). Respondents were then read a list of nine symptoms and asked whether they thought that each symptom could be a sign of cancer: unexplained lump or swelling, persistent unexplained pain, unexplained bleeding, persistent cough or hoarseness, persistent change in bowel or bladder habits, persistent difficulty swallowing, change in the appearance of a mole, sore that does not heal and unexplained weight loss), each with response options of ‘Yes, ‘No' and ‘Don't know'. We examined change in recall and recognition of the three symptoms that were closest to those targeted in the campaign: ‘cough' or ‘hoarseness', ‘change in bowel or bladder habits' and unexplained bleeding (this was counted as correct whether or not there was mention of rectal bleeding, because the coding system used by the ONS did not specify the source of bleeding). The other six symptoms served as control symptoms.

Perceived barriers to seeking help were assessed using the following question: ‘Sometimes people put off going to see the doctor, even when they have a symptom they think might be serious. Could you say if any of these might put you off going to the doctor?' This was followed with a list of 10 potential barriers: too embarrassed, too scared, worried about what the doctor might find, not feeling confident talking to the doctor, being too busy, having too many other things to worry about, difficulty arranging transport, worried about wasting the doctor's time, the doctor would be difficult to talk to and difficulty making an appointment with the doctor, with response options of ‘Yes, often', ‘Yes, sometimes' and ‘No'. We examined change in the frequency of the two barriers closest to those targeted in the campaign (worry about wasting the doctor's time, the doctor being difficult to talk to). The other eight were treated as control items.

Cancer experience was measured with the item ‘Have you, or any of your friends or family, had cancer? With response options: ‘you', ‘your partner', ‘close family member', ‘other family member', ‘close friend', ‘other friend', ‘none of these' and ‘prefer not to say'. This item was dichotomised to distinguish respondents with some *vs* no experience of cancer.

The Opinions and Lifestyle survey provides a range of social demographic variables. The following were included in this analysis: gender (male, female), age (16–24, 25–34, 35–44, 45–54, 55–64, 65 years and over), marital status (married/civil partnership/cohabiting, not married), ethnicity (White, other ethnic backgrounds), highest education qualification (degree or equivalent, below degree, other, no formal qualifications) and occupation (managerial/professional, intermediate/small employers/lower supervisory, semi-routine/routine) which served as an indicator of socioeconomic status.

### Analyses

Differences between survey years in recall and recognition of the target symptoms and the barriers addressed by the campaigns were tested using logistic regressions. Multivariable logistic regression was used to examine differences in symptom recall and recognition and help-seeking barriers controlling for demographic differences between the two surveys. Secondary analyses investigated interactions between survey year and gender, age, ethnicity, occupation and cancer experience.

## Results

In 2010, the response rate was 58% (2090/3601), with 1170 (32%) refusals, 34 (1%) with unknown eligibility and 307 who could not be contacted after three attempts (9%). In 2012, the response rate was 55% (2001/3667), with 1177 (32%) refusals, 21 (<1%) with unknown eligibility and 468 (12.8%) who could not be contacted after three attempts. There were no differences in gender or age between years, but 2012 respondents were less likely to be married, more likely to be from a non-White ethnic group, were from higher status occupational groups, fewer had no educational qualifications and were more likely to have experience of cancer (see Table 1).

### Recall of symptoms

Recall of ‘cough' or ‘hoarseness' significantly increased from 18% in 2010 to 26% in 2012 (*P*<0.001). Recall of ‘change in bowel or bladder habits' doubled from 21% in 2010 to 43% in 2012 (*P*<0.001), but the increase in recall of unexplained bleeding (31%–33%, *P*=0.282) was not significant. Recall of other signs and symptoms (e.g., lump, pain, difficulty swallowing and change in mole) was similar in the two surveys, except for a decrease in recall of unexplained weight loss as a symptom (27% to 22%, *P*<0.001) (see Table 2).

Using multivariable logistic regression adjusting for demographic variables, the differences in recall of ‘cough or hoarseness' remained significant (*β*=1.67 (CI: 1.41–1.98) *P*<0.001), as did recall of ‘change in bowel or bladder habits' (*β*=2.86 (CI: 2.44–3.36) *P*<0.001). The reduction in weight loss was borderline significant (*β*=0.79 (CI: 0.67–0.94), *P*=0.013).

There were no significant interactions between survey year and gender, age, ethnicity, occupation or cancer experience in the changes in symptom recall.

### Recognition of symptoms

Recognition of symptoms was much higher across the board than recall, but nonetheless significant increases in awareness were only apparent for the three target symptoms (persistent cough or hoarseness, change in bowel or bladder habits and unexplained bleeding). Recognition that a persistent cough or hoarseness could be a sign of cancer increased from 67% in 2010 to 78% in 2012 (*P*<0.001). Recognition of a change in bowel or bladder habits increased from 87% to 91% (*P*<0.001), and there was a small increase in unexplained bleeding from 84% to 87%, which was borderline significant (*P*=0.014) (see Table 2). There were no significant changes in recognition of other symptoms.

Differences for persistent cough or hoarseness (*β*=1.88 (CI: 1.58–2.23) *P*<0.001) and change in bowel or bladder habits habits (*β*=1.47 (CI: 1.14–1.88) *P*=0.003) remained significant after controlling for demographic differences between the samples, but did not for unexplained bleeding (*β*=1.20 (CI: 0.97–1.48) *P*=0.086).

There were no significant interactions between survey year and gender, age, ethnicity, occupation or cancer experience in the changes in symptom recognition.

### Perceived barriers to seeking medical help

There was no significant change in being ‘worried about wasting the doctor's time' (down from 26% in 2010 to 24% in 2012, *P*=0.158) or believing that the ‘doctor would be difficult to talk to' (14% in 2010 and 13% in 2012, *P*=0.617), and this did not change in multivariate analyses controlling for demographics. However, other barriers, such as being ‘too scared', being ‘worried about what the doctor might find', ‘having too many other things to worry about' and ‘difficulty arranging transport', which were not directly targeted in the campaign, were significantly less likely to be endorsed in 2012 compared with 2010 (see Table 3). In multivariate analyses being ‘too scared' (*β*=0.79 (CI: 0.66–0.95) *P*=0.016), ‘being worried about what the doctor might find' (*β*=0.70 (CI: 0.67–0.92) *P*=0.002) and ‘difficulty arranging transport' (*β*=0.50 (0.32–0.78) *P*=0.002) remained significant. ‘Having too many other things to worry about' was no longer significant (*β*=0.86 (0.71–1.05) *P*=0.129), and the increase in ‘being too busy' (*β*=1.26 (1.06–1.49) *P*=0.009) became significant.

## Discussion

These results suggest that the national BCOC campaigns on lung and bowel cancer were associated with increased public awareness of the symptoms targeted by the campaigns. Recall and recognition of a persistent cough as a potential sign of cancer increased markedly, indicating that the lung campaign had an enduring impact on knowledge given that the advertising stopped 3–4 months before the 2012 survey. These findings echo those from the lung campaign evaluation, which also demonstrate that the campaign was associated with increases in urgent GP referrals, the number of lung cancers diagnosed and the proportion of early-stage lung cancers (Ironmonger *et al*, 2015).

The proportion of people freely recalling change to bowel or bladder habits as a potential symptom of cancer doubled between 2010 and 2012, and recognition of these symptoms from a prompted list also significantly increased. Neither recall nor recognition of unexplained bleeding improved between surveys. This is probably because it was not possible to distinguish mentions of rectal bleeding (one of the key symptoms highlighted in the BCOC bowel campaign) and all other mentions of bleeding, which obscured any impact. The larger increase in awareness of bowel symptoms is likely owing to the timing of surveys because the national bowel campaign was repeated at a similar time to the 2012 survey.

Improvements in awareness occurred in both men and women, across all levels of occupation and education and in White and non-White ethnic groups. Campaign evaluation shows similar findings with improvements in recall evident among men and women and different social groups (Moffat *et al*, 2015). This is encouraging because awareness of cancer signs and symptoms tends to be higher among women, people from more affluent backgrounds and White ethnic groups (Robb *et al*, 2009). In addition, health information campaigns sometimes show a greater impact on more affluent groups (Berkman *et al*, 2011), with the potential to increase existing inequalities (Viswanath, 2005).

In contrast to the effects on awareness of symptoms, the campaigns appeared to have little impact on beliefs about GP approachability, despite doctors featuring prominently in the adverts. There are a few potential explanations for this. People may not relate to the doctors featured in the campaigns because they were not their own GP. More personalised communications may have a greater impact. For example, letters sent directly from GPs endorsing the bowel cancer screening programme have significantly improved uptake (Hewitson *et al*, 2011). Future research could test whether this type of approach can change perceptions about GP approachability. The communications about the doctor were less direct than those about symptoms and perhaps too subtle to have made an impact. More in-depth qualitative feedback would help understand whether this is the case. Attitudes are also notoriously difficult to change, and it may be too early to see any impact as a result of these campaigns.

It was interesting that ‘being worried about what the doctor might find' and ‘being too scared' significantly reduced between 2010 and 2012, given that neither campaign addressed worry or fear specifically. It is possible this reflects a trend towards more positive attitudes about cancer, but longer-term tracking will be needed to explore this further.

Believing having difficultly arranging transport would be a barrier to seeing the doctor also significantly reduced between 2010 and 2012 surveys. This is surprising given that only 5% of the respondents thought that this might put them off seeing the doctor in 2010. Again, longer-term tracking is needed to help us understand this change.

There is now evidence from the evaluation of the bowel and lung campaigns, which shows that the campaigns were associated with an increase in GP attendances for symptoms directly linked to the campaigns (Moffat *et al*, 2015). Given that this appears to have occurred without any change in the perceived approachability of the GP, it suggests that other barriers to seeking help, which we did see reduce, such as being worried about what the doctor might find, may be playing a role. It could also be a direct effect of the raised symptom awareness. This is consistent with evidence that not appreciating the seriousness of symptoms is a common explanation for delayed symptomatic presentation (Macleod *et al*, 2009). It is also consistent with evidence that differences in symptom awareness across the population are associated with differences in anticipated delay before visiting the doctor (Robb *et al*, 2009; Quaife *et al*, 2014).

Moffat *et al* (2015) found that the increase in GP attendances associated with both campaigns was larger among those aged 50–59 years. For the bowel campaign, increases were greater among men and within practices in the most-deprived areas, whereas the increase in attendances associated with the lung campaign were significantly greater for practices in less-deprived areas. Coupled with our results, this could indicate that although the campaigns have been associated with increased symptom awareness across all population groups, they may have been more successful in prompting action among older groups specifically (aged 50–59 years). Continuing to monitor and explore the impact of the campaigns on awareness, beliefs and behaviour across population groups will help determine whether this is the case and ensure that the campaigns are being appropriately targeted.

This study benefits from data collection by the ONS Opinions and Lifestyle survey, which uses ‘gold standard' methods for recruiting a nationally representative sample of Great Britain, maximising generalisability. Using a validated measure to assess awareness and barriers ensures reliable responses that are directly comparable over time (Stubbings *et al*, 2009). CAM data also provide evidence of changes in recall, as well as recognition, both of which may be important. Recall may provide a more robust indication of knowledge, although it has been argued that recognition is more relevant to the scenario of experiencing a symptom and recognising its significance. Limitations include the reliance on hypothetical questions about barriers to visiting the GP, and the assumption that people will be aware of the key barriers in their own situation. Comparisons between pre- and post-campaigns were made using existing data, and therefore the timing of the surveys was not ideal, and it was not possible to determine respondents' exposure to the campaigns. To the best of our knowledge, the lung and bowel BCOC campaigns were the only national media campaigns run during the study period that aimed to raise awareness of the targeted symptoms and address beliefs about GP approachability. However, other factors may have contributed to increased awareness that were not controlled for here. Although response rates were good (⩾55%), the resulting sample was self-selected and therefore may not be representative of the British public. Lastly, respondents were informed that they would be answering questions about cancer awareness on behalf of Cancer Research UK, and this could have influenced their responses to the attitudinal items regarding potential barriers to visiting the doctor.

In conclusion, the results indicate that the BCOC national lung and bowel campaigns have been associated, and encourage people with relevant symptoms to visit their GP, have been associated with improved public awareness of campaign-targeted symptoms across all population groups. A different approach may be needed to achieve changes in public perceptions of GP approachability, and interventions that target primary care in addition to the public may also be needed. It will be important to monitor awareness and beliefs over the longer term and on a regular basis. This will help focus future funding in resources and interventions and ensure that they have maximum impact.

## Figures and Tables

**Table 1:** Demographic characteristics of the survey samples

	**2010 (*****n*****=2090)**	**2012 (*****n*****=2001)**
**Gender**		
Male	44.8 (937)	45.5 (910)
Female	55.2 (1153)	54.5 (1091)
**Age (years)**		
16–24	7.1 (149)	8.1 (163)
25–34	13.3 (277)	14.8 (297)
35–44	17.5 (366)	15.6 (312)
45–54	16.8 (352)	16.3 (327)
55–64	18.1 (379)	17.4 (348)
65–74	14.7 (308)	15.2 (305)
75 and over	12.4 (259)	12.4 (249)
**Marital status**		
Married/civil partnership	46.1 (963)	43.3 (866)*
Not married	53.9 (1127)	56.7 (1135)
**Ethnicity**		
White	92.9 (1941)	91.4 (1828)*
Other ethnic backgrounds	7.1 (149)	8.6 (173)
**Occupation**		
Managerial/professional (higher SES)	36.8 (688)	42.1 (576)*
Intermediate/small employers/lower supervisory (mid-SES)	21.6 (404)	26.3 (359)
Semi-routine/routine (lower SES)	41.6 (779)	31.6 (432)
**Highest qualification obtained**		
Degree or equivalent	18.5 (387)	22.6 (452)*
Below degree	40.2 (839)	43.9 (878)
Other	15.0 (313)	12.1 (242)
No formal qualifications	26.3 (550)	21.4 (429)
**Cancer experience**		
Some	82.3 (1684)	86.6 (1686)*
None	17.7 (362)	13.4 (261)

Abbreviation: SES=socioeconomic status.

Some variables do not add up to 100% owing to missing data.

*Significant difference *P*<0.05.

**Table 2:** Recall and recognition of warning signs/symptoms

	**2010** **% (*****n***)	**2012** **% (*****n***)	**Odds ratio (95% CI)**	**Adjusted odds ratio (95% CI)**	***P***
**Recall**					
**Target symptoms**					
Persistent cough or hoarseness	18.2 (380)	26.1 (523)	1.59 (1.37–1.85)	1.67 (1.41–1.98)	0.000
Persistent change to bowel/bladder habits	20.9 (437)	42.5 (850)	2.79 (2.43–3.21)	2.86 (2.44–3.36)	0.000
Unexplained bleeding	31.2 (653)	33.2 (664)	1.09 (0.96–1.25)	1.09 (0.93–1.27)	0.282
**Other symptoms**					
Unexplained lump	69.0 (1443)	68.4 (1368)	0.97 (0.85–1.11)	0.92 (0.78–1.08)	0.320
Persistent unexplained pain	24.4 (510)	25.5 (511)	1.06 (0.92–1.22)	0.97 (0.82–1.15)	0.730
Persistent difficulty swallowing	4.5 (93)	4.8 (96)	1.08 (0.81–1.45)	1.15 (0.83–1.60)	0.299
Change in the appearance of a mole	25.2 (527)	23.6 (473)	0.92 (0.80–1.06)	0.94 (0.80–1.11)	0.492
Sore that does not heal	4.7 (99)	6.3 (126)	1.35 (1.03–1.77)	1.37 (0.99–1.88)	0.055
Unexplained weight loss	27.3 (571)	21.8 (436)	0.74 (0.64–0.86)	0.79 (0.67–0.94)	0.013
**Recognition**					
**Target symptoms**					
Persistent cough or hoarseness	67.4 (1393)	78.1 (1551)	1.73 (1.50–1.99)	1.88 (1.58–2.23)	0.000
Persistent change to bowel/bladder habits	86.8 (1794)	90.6 (1799)	1.47 (1.21–1.79)	1.47 (1.14–1.88)	0.003
Unexplained bleeding	83.8 (1732)	86.5 (1718)	1.24 (1.05–1.48)	1.20 (0.97–1.48)	0.086
**Other symptoms**					
Unexplained lump	93.0 (1923)	92.7 (1842)	0.97 (0.76–1.23)	1.02 (0.75–1.38)	0.913
Persistent unexplained pain	73.6 (1523)	71.2 (1414)	0.89 (0.77–1.02)	0.83 (0.70–0.98)	0.027
Persistent difficulty swallowing	75.2 (1556)	74.0 (1469)	0.94 (0.81–1.08)	0.92 (0.77–1.09)	0.322
Change in the appearance of a mole	93.1 (1926)	94.0 (1867)	1.16 (0.90–1.49)	1.04 (0.74–1.47)	0.813
Sore that does not heal	58.9 (1218)	57.6 (1144)	0.85 (0.84–1.07)	0.87 (0.75–1.01)	0.069
Unexplained weight loss	85.0 (1758)	83.1 (1650)	0.87 (0.73–1.03)	0.86 (0.70–1.07)	0.257

Abbreviation: CI=confidence interval.

**Table 3:** Barriers to help-seeking

	**2010**	**2012**	**Odds ratio (95% CI)**	**Adjusted odds ratio (95% CI)**	***P***
**Target barriers**					
Wasting time	26.1 (537)	24.2 (476)	0.90 (0.78–1.04)	0.86 (0.73–1.02)	0.075
Difficult to talk to	13.9 (277)	13.3 (255)	0.95 (0.80–1.15)	0.98 (0.79–1.22)	0.857
**Other barriers**					
Embarrassed	17.4 (356)	15.4 (303)	0.87 (0.73–1.02)	0.85 (0.70–1.04)	0.117
Scared	23.2 (473)	19.7 (385)	0.81 (0.70–0.94)	0.79 (0.66–0.95)	0.016
Worried	37.1 (756)	32.1 (630)	0.80 (0.70–0.91)	0.70 (0.67–0.92)	0.002
Lack of confidence	9.6 (196)	11.4 (225)	1.22 (1.00–1.50)	1.23 (0.96–1.57)	0.096
Transport	5.1 (106)	3.6 (72)	0.70 (0.51–0.95)	0.50 (0.32–0.78)	0.002
Difficulty making an appointment	36.5 (746)	39.0 (760)	1.11 (0.98–1.26)	1.19 (1.02–1.38)	0.025
Too busy	21.1 (434)	21.9 (432)	1.05 (0.90–1.22)	1.26 (1.06–1.49)	0.009
Other priorities	17.5 (359)	14.6 (288)	0.81 (0.68–0.96)	0.86 (0.71–1.05)	0.129

Abbreviation: CI=confidence interval.
